# Heroin Hydrochloride as an Analgesic

**Published:** 1900-04

**Authors:** 


### Abstracts.

### Heroin Hydrochloride as an Analgesic.

Dr. Kurt Witthauer (*Die Heilkunde*, February, 1900,) details his experience with heroin hydrochloride which he administered in solution with aq. laurocerasi. His most favorable results with this drug were obtained in the treatment of simple tracheitis. He has also found it of distinct value in laryngitis, pleuritis, pneumonia, in the first stage, and in the dry forms of pulmonary tuberculosis. It is advisable to commence with small doses of 1-24 grain, and then increase the dose to 1-12 grain, three times daily; larger doses are seldom required. As an analgesic heroin hydrochloride was successfully employed in pleurisy with lancinating pains, for the relief of pains in ovaritis, and also for the gastric distress of nervous dyspepsia. Satisfactory results were also obtained from the subcutaneous use of the drug in a case of sciatica, and in transverse myletis with painful contractures of the legs. In the last stage of miliary tuberculosis, with constant cough and stabbing pains, heroin hydrochloride completely replaced the hypodermatic injection of morphine. In all of these cases the initial dose was 1-24 grain, which was increased to 1-12 grain, if necessary. In several instances of myocarditis, with feelings of anxiety and dyspnea, the drug exerted a sedative influence.

